# Correlation between the progression of diabetic retinopathy and inflammasome biomarkers in vitreous and serum – a systematic review

**DOI:** 10.1186/s12886-022-02439-2

**Published:** 2022-05-27

**Authors:** Charisse Y. J. Kuo, Rinki Murphy, Ilva D. Rupenthal, Odunayo O. Mugisho

**Affiliations:** 1 Buchanan Ocular Therapeutics Unit, New Zealand National Eye Centre, Department of Ophthalmology, The University of Auckland, Auckland, 1023 New Zealand; 2 Department of Medicine, Faculty of Medical and Health Sciences, The University of Auckland, Auckland, 1023 New Zealand

**Keywords:** Diabetic retinopathy, Inflammasome, Biomarkers, Vitreous, Serum, Cytokines

## Abstract

Activation of the NOD-like receptor protein 3 (NLRP3) inflammasome pathway has been implicated in Diabetic retinopathy (DR) pathogenesis, but its impact on DR development and progression remains unclear. Therefore, the primary aim of this systematic literature review was to determine the role of the inflammasome in DR development. Furthermore, the secondary aim was to determine whether systemic inflammasome activity can be used to predict DR progression. Studies measuring vitreous and/or serum inflammasome biomarkers in DR patients with Type 2 Diabetes Mellitus (T2DM) were searched systematically using online databases EMBASE, PubMed and Web of Science with the last search conducted on 29^th^ of September 2021. The risk of bias was assessed using the Newcastle Ottawa Scale and 20 studies were eligible for narrative analysis. Limitations included the heterogeneity in detection assays used, the small and uneven sample size, a lack of vitreous data in earlier disease stages, and not accounting for patients with other systemic co-morbidities. Analysis showed that inflammasome biomarkers IL-1β and IL-18 increased significantly from non-proliferative DR to proliferative DR in both vitreous and serum, suggesting the inflammasome pathway is activated as DR progresses and that serum inflammasome levels could be explored as potential biomarkers for DR progression.

**Supplementary Information:**

The online version contains supplementary material available at 10.1186/s12886-022-02439-2.

## Introduction

Diabetic retinopathy (DR) is the leading cause of preventable blindness in the working-age population worldwide [1]. In 2010, DR was prevalent in approximately one third of the diabetic population, affecting over 90 million people globally [2]. The number of patients with DR is set to increase in parallel with the continuous rise in the prevalence of diabetes, amplifying the burden on healthcare systems and the economy [1]. Therefore, it is imperative to provide a preventative treatment for this sight-threatening disease.

DR is characterized by the loss of retinal vascular integrity, which results in the development of vascular lesions including microaneurysms, dot and blot hemorrhages and hard exudates [3–5]. Proliferative diabetic retinopathy (PDR) occurs at the late disease stage when increased oxygen demand due to retinal ischemia drives the formation of new but fragile and leaky vessels (neovascularization), which can lead to sight-threatening complications such as hemorrhages in the vitreous and retinal detachment [3, 4]. Furthermore, diabetic macular edema (DME), which involves fluid retention in the macular region of the retina, can cause reduced vision at any stage of DR [3–5].

Several treatment options are available for DR and DME, including pan-retinal photocoagulation, intravitreal anti-vascular endothelial growth factor (anti-VEGF) injections and intravitreal corticosteroids [6]. However, they are only administered when the retina is already inflamed. Other limitations include high treatment costs, high prevalence of non-responders, requirement for repeated injections and increased resistance to treatment with repeated administration or side effects such as development of geographic atrophy and ocular hypertension [7–9]. Furthermore, clinical studies have demonstrated that anti-VEGF agents do not target the underlying DR pathogenesis and therefore do not prevent DR onset, relapse, and further progression [4, 7]. Maturi et al*.*, 2021 [10] reported that despite reducing DME and PDR by almost three folds relative to sham, anti-VEGF treatment did not improve visual acuity and did not fully prevent DR progression. Baker et al*.*, 2019 [11] also showed no significant difference in the reduction of visual acuity at two years between those who received anti-VEGF treatment and those only being monitored for progression, implying that anti-VEGF agents do not prevent vision loss in the long term. More recently, advanced imaging techniques such as optical coherence tomography (OCT) and ultra-widefield imaging are also increasingly being used in DR management as they provide detailed information regarding the severity of peripheral ischemia and changes in retinal and choroidal vasculature [6]. Other clinical approaches such as glucose and blood pressure control, as well as smoking cessation, are used to reduce the risk of DR development. However, DR can still develop even in the presence of these measures, suggesting that the key mechanism required for DR onset and progression is not being targeted by the current approaches [12–15]. Management of currently known risk factors (high blood glucose, blood pressure, lipids) is insufficient for controlling DR progression, therefore identification of biomarkers that can accurately predict DR progression is urgently required.

The pathogenesis correlating chronic systemic hyperglycemia with the development of vascular lesions in DR is not fully elucidated. In contrast to previously proposed mechanisms which reflect DR as a single hyperglycemia-induced process, including formation of advanced glycation end products as well as the polyol, protein kinase C and hexosamine pathways, recent studies have demonstrated that diabetic complications, including DR, are underpinned by a complex interplay between metabolic and inflammatory changes [16, 17]. This process is thought to be facilitated by the activation of the NOD-like receptor protein 3 (NLRP3) inflammasome, a part of the innate immune system that orchestrates inflammatory cascades in response to cellular stress signals, which becomes dysregulated and aggravates chronic inflammation in DR [16, 18–20]. Upon stimulation, the NLRP3 protein aggregates with apoptosis-associated speck-like protein containing a CARD (ASC), and procaspase-1, leading to the autolytic cleavage and activation of procaspase-1, subsequently releasing pro-inflammatory cytokines IL-1β and IL-18 into their active forms which mediate downstream inflammatory cascades in DR [21–23]. IL-1β plays several roles in DR vascular lesions, including accelerating apoptosis of endothelial and Müller cells, as well as augmenting the activity of transcription factor NFκB, leading to elevated levels of pro-inflammatory cytokines such as IL-6 and TNF-α [24, 25]. Furthermore, IL-1β can auto-stimulate to amplify its own production, or work in tandem with VEGF to upregulate each other’s expression levels [26, 27]. On the other hand, the role of IL-18 in DR remains enigmatic as it can either promote or suppress angiogenesis [26]. Some studies have suggested that IL-18 is pro-angiogenic as it upregulates chemokines and pro-inflammatory cytokines which promote leukocyte activation, angiogenesis, endothelial cell migration and tubule formation. In contrast, others have demonstrated that IL-18 is anti-angiogenic, as it provides protection against VEGF-induced retinal leakage, retinal and sub-retinal neovascularization, tight junction disruptions, and laser-induced choroidal neovascularization [28–32]. Through inhibiting the NLRP3 inflammasome pathway either directly or indirectly, key molecular and clinical DR signs, such as the release of pro-inflammatory cytokines, oedema, neuronal death, and vascular leakage in the retina, can be prevented [25, 33–37]. As such, the NLRP3 inflammasome pathway, mainly through the action of IL-1β, is a potential therapeutic target for preventing the onset and progression of DR, while serum inflammasome markers could also act as a potential biomarker of DR onset and progression.

Whilst these studies highlight the importance of the inflammasome pathway in DR, the direct or indirect impact of the inflammasome on DR onset and progression has not been investigated extensively. Therefore, the primary aim of this systematic literature review is to determine the role of the inflammasome in DR development and progression by correlating serum and vitreous inflammasome levels with DR progression. The secondary aim is to determine whether systemic inflammasome activity can be used to predict DR progression by testing whether higher inflammasome activity is associated with more rapid DR progression. This systematic literature review suggests a close association between the development and progression of DR and vitreous inflammasome biomarkers. Increased levels of inflammasome biomarkers were also found in the serum of DR patients with more advanced DR compared to those at early stages of the disease, suggesting that blood tests could potentially be used clinically to predict the development and progression of DR. This study provides further insight into the role of the inflammasome in DR pathogenesis, and opens up opportunities for the development of new drugs which specifically target the NLRP3 inflammasome pathway.

## Results

### Study selection

**Fig. 1:**
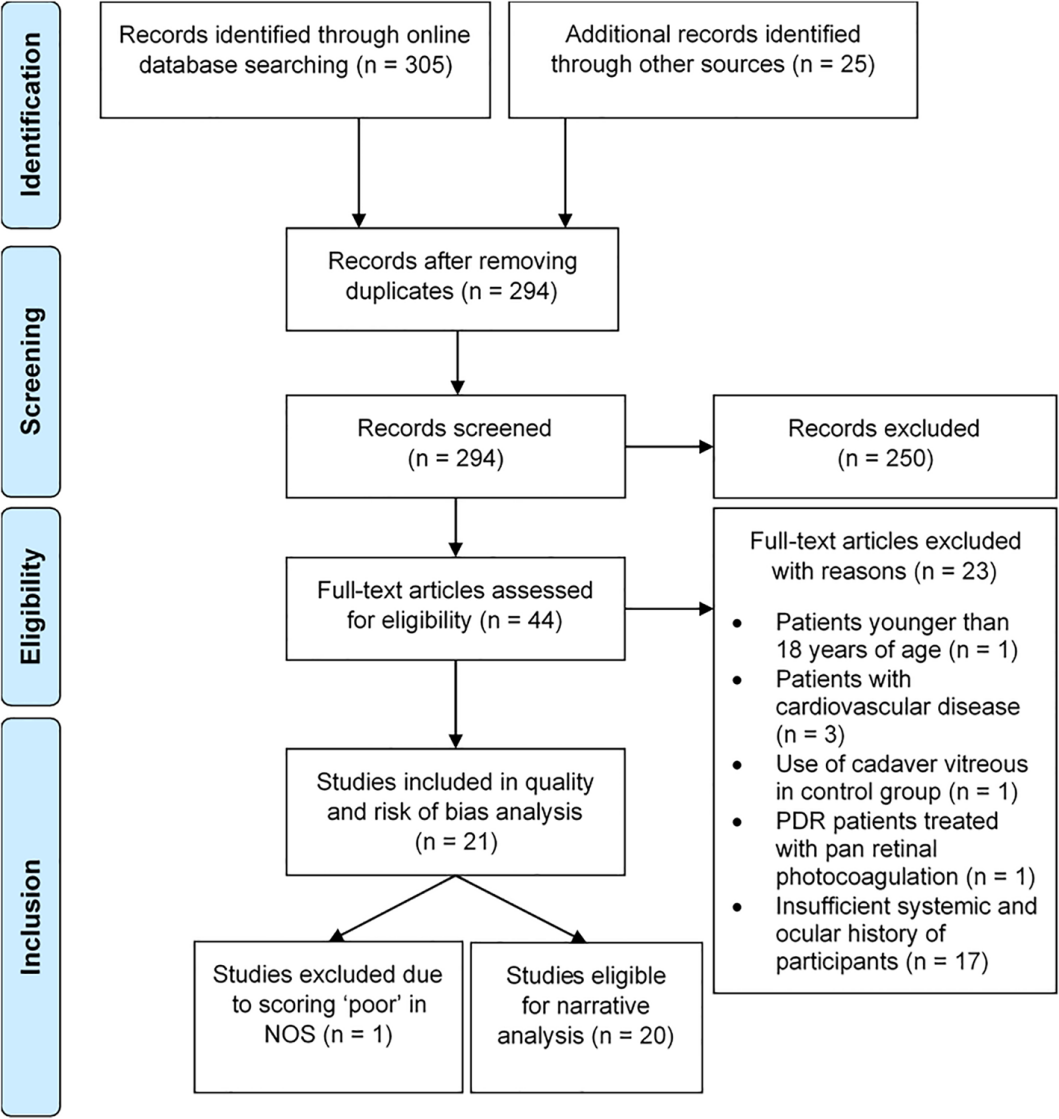
PRISMA flowchart illustrating the study selection process

Using pre-specified keywords in the search strategy (Table S1), 305 studies were identified from online databases EMBASE, PubMed and Web of Science, with an additional 25 studies identified through study references. Duplicated studies, animal and in vitro studies, conference abstracts and review articles, as well as manuscripts not written in English were excluded. The full texts of the remaining 44 studies were assessed and 23 were subsequently excluded due to participants not meeting the inclusion criteria (Table 1). Finally, 21 studies were assessed for quality and risk of bias assessment. The selection process is outlined in the PRISMA flowchart (Fig. 1).

**Table 1:** PICO criteria for the inclusion of studies

*Population*	Type 2 Diabetes Mellitus (T2DM) patients with DR
*Intervention*	Not applicable as only observational studies were included
*Control*	Non-diabetic patients without DR; in studies where non-diabetic patients were not recruited, T2DM patients without DR was used as the control group
*Outcomes*	Association between DR progression and inflammasome biomarkers in vitreous and serum

### Quality and risk of bias

Quality and risk of bias were assessed using the predefined criteria and scoring system in the modified Newcastle Ottawa Scale (NOS) (Table S2), which identified seven good, 13 fair and one poor study. The study rated as “poor” [38] was excluded from further analysis as it did not specifically exclude patients with systemic inflammatory diseases. Therefore, 20 studies were eligible for narrative analysis.

### Clinical profiles

The clinical profiles of participants in the included studies are summarized in Table S3. All participants were age and gender matched for all study groups, except for Blum et al*.*, 2018 [39], which had a significantly younger control group. The participant age in the majority of studies ranged between 50 and 70, except for the control group of Blum et al*.*, 2018 [39] (36.6 ± 7.9) and Kaviarasan et al*.*, 2015 [40] (44 ± 7), the NPDR group of Blum et al*.*, 2018 [39](71.4 ± 8.9) and the diabetic groups of Cvitkovic et al*.*, 2020 [41] (71.9 ± 6.7 in NDR, 73.2 ± 5.1 in DR). In general, patients with more severe DR had a longer duration of diabetes, and a higher HbA1c%. However, clinical profiling revealed that crucial information required for cross analysis was not available (Table S3).

### Study characteristics

Of the 20 eligible studies, 19 were cross-sectional case–control [39–58] and one was longitudinal [59]. In total, eight studies [40, 43, 44, 50, 52, 53, 55, 57] graded DR severity using the International Clinical Diabetic Retinopathy Disease Severity Scale (ICDRDSS), four [46, 48, 49, 54] used the Early Treatment Diabetic Retinopathy Study Scale (ETDRSS), five [39, 45, 47, 51, 59] relied on the ophthalmologists and the remaining three studies [41, 42, 58] did not report the grading method (Table S4). T2DM patients were grouped according to the DR severity level: no DR (NDR), non-proliferative DR (NPDR) and proliferative DR (PDR). Two studies measured only vitreous [43, 58], 15 studies [39, 41, 44–55, 59] measured only serum and three studies [40, 42, 57] determined both serum and vitreous biomarker levels. Moreover, different assays were employed to measure the biomarker levels in serum and vitreous. For studies only using one biomarker detection assay, 10 [39, 42, 43, 45, 47, 48, 50, 52, 57, 58] employed enzyme-linked immunosorbent assay (ELISA), three [44, 53, 55] used a Luminex magnetic assay, two [41, 54] performed cytometric bead array (CBA), and two [46, 49] utilized a chemiluminescent immunometric assay (Table S4). Furthermore, three studies [40, 51, 59] used two assays to detect different biomarkers (Table S4). Due to the large heterogeneity between grading scales, detection assays and biomarkers of interests, results were not suitable for meta-analysis.

### General biomarker levels

#### Pro-inflammatory biomarkers

Overall, studies found a significant increase in pro-inflammatory biomarkers in both the vitreous and serum of patients with PDR compared to controls, with IL-6 and TNF-α being the most studied biomarkers (Table 2). In total, 10 serum pro-inflammatory biomarkers increased significantly in PDR relative to NPDR (IL-6: [40, 41, 44], TNF-α: [46, 48, 51, 54], CRP: [51], NO: [46] IL-2R: [46], sIL-6R: [44], IL-12: [47], VEGF: [48, 51]) IL-1β: [48], IL-18: [47]).

**Table 2:** Studies measuring vitreous and serum biomarker levels in DR patients compared to controls

		*Vitreous*			*Serum*		
	Biomarker	Increased	Not changed	Decreased	Increased	Not changed	Decreased
*Pro-inflammatory*	VEGF	[40, 58]			[48, 51, 53]	[40, 45, 49]	
	IL-1β	[43, 58]			[41, 48]	[49] [54]	
	IL-18	[43]			[47]		
	TNF-α	[42, 58]	[40]		[41, 42, 46, 48, 51, 59] [54]	[40, 49]	
	ET-1	[58]					
	IL-6	[40, 42, 58]			[40–42, 44, 48, 49, 54, 59]		
	IL-4		[40]			[40, 49] [54]	
	IL-2		[40]			[40, 49]	
	IFN-*γ*		[40]		[41]	[40, 49]	
	IL-2R				[46]		
	sIL-6R				[44]		
	sgp130				[44]		
	NO				[46]		
	IL-17A				[54, 54]		
	IL-1α					[49]	
	MIP-1α					[53]	
	CRP				[51, 59]		
	IL-12				[41, 47]		[54]
*Anti-inflammatory*	BNDF			[40]			[40]
	LXA4			[40]			[40]
	IL-10	[40]	[58]		[41]	[40, 49]	
	PEDF	[40]			[52]	[40]	
	IL-22					[55]	
	IL-27		[57]				[57]
	IL-35			[57]			[57]
*Chemotactic*	VCAM-1	[42]			[39, 42]		
	ICAM-1	[42]			[42]		
	MCP-1/ CCL2	[58]			[50, 53]	[41, 49]	
	IL-8	[58]			[41, 46, 49]	[53, 54]	
	sE-selectin				[39]		
	IP-10				[47]		
*Cell growth*	IGF-1						[45]
	EGF					[49, 53]	

*BDNF* brain derived neurotrophic factors, *CRP* C reactive protein, *EGF* epidermal growth factor, *ICAM-1* intercellular adhesion molecule-1, *IFN-γ *interferon gamma, *IGF-1* Insulin-like growth factor 1, *IL* interleukin, *IL-1Rα* Interleukin-1 receptor alpha, *IP-10* Interferon-Inducible Protein 10, *LXA4* lipoxin A4, *MIP-1α* macrophage inflammatory protein, *MCP-1/CCL2* monocyte chemoattractant protein-1/chemokine (C–C motif) ligand 2, *NO* nitric oxide, *PEDF* pigment epithelium derived factor, *sgp130* soluble glycoprotein 130, *TNF-α* tumor necrosis factor-alpha, *VCAM-1* vascular cell adhesion molecule-1, *VEGF* vascular endothelium growth factor

#### Anti-inflammatory biomarkers

Studies did not reveal any changes in vitreous anti-inflammatory biomarker levels in PDR patients compared to controls. In PDR compared to control, Kaviarasan et al*.*, 2015 [40] found significantly reduced BNDF and LXA4 in both vitreous and serum, while IL-10 and PEDF were increased in the vitreous but unchanged in the serum. In contrast, Zhou et al*.*, 2012 [58] found no significant difference in vitreous IL-10 between PDR and control, while Cvitkovic et al*.*, 2020 [45] found a significant increase in serum IL-10 in NDR and DR relative to control, and Ogata et al*.*, 2007 [52] found significantly higher plasma levels of PEDF in PDR patients compared to control (Table 2).

#### Chemotactic biomarkers

Studies showed an overall increase in chemotactic biomarker levels, including VCAM-1, ICAM-1, MCP-1/CCL2 and IL-8, in the vitreous and serum of DR patients compared to controls (Table 2). A total of five chemotactic biomarkers were significantly increased in PDR relative to NPDR (VCAM-1: [39], MCP-1/CCL2: [53], IL-8: [46], sE-selectin: [39], IP-10: [47]).

### Comparison between vitreous and systemic biomarker levels

**Fig. 2:**
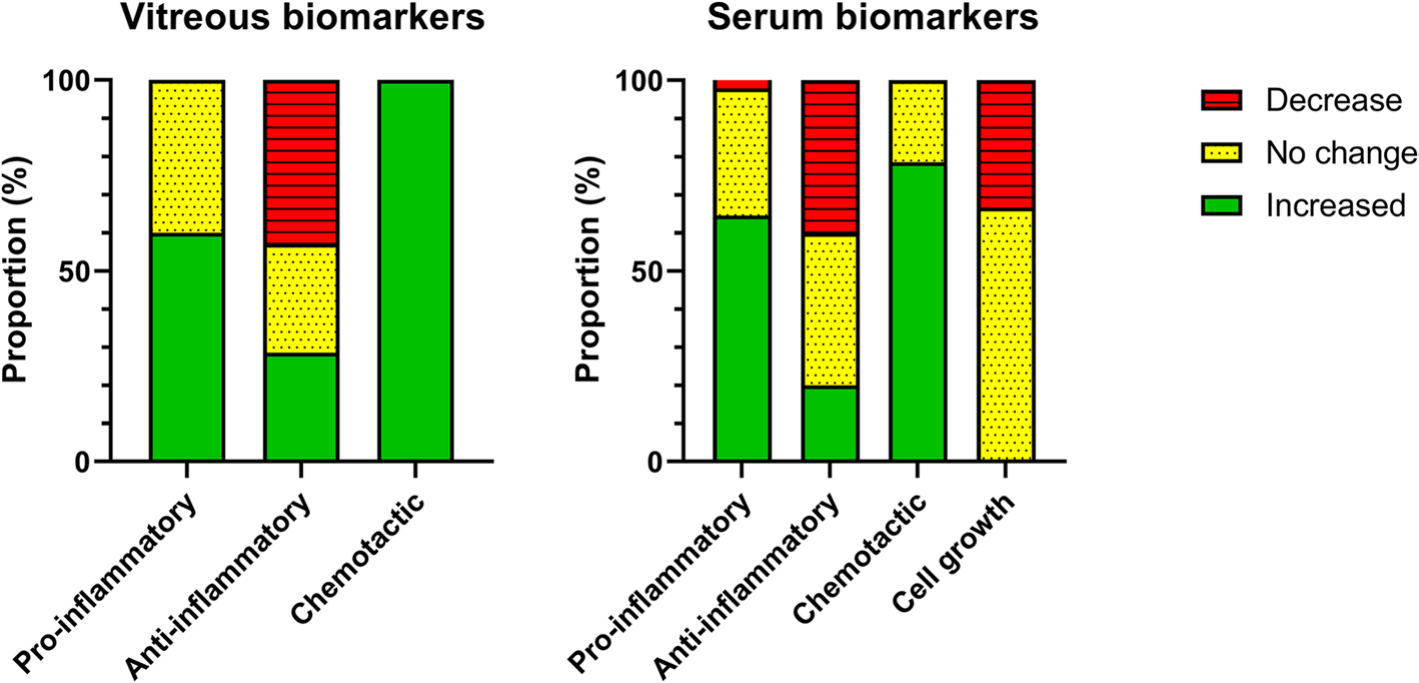
Proportion of studies showing changes in vitreous and serum biomarker levels in DR patients compared to controls

The proportion of studies reporting an increase, no change, or a decrease in the levels of vitreous and serum biomarkers in DR patients relative to controls are illustrated in Fig. 2. A similar proportion of studies reported an increase or no change in pro-inflammatory biomarkers in the vitreous (increase: 60%; no change: 40%) and serum (increase: 64.6%; no change: 33.3%) relative to controls. Similarly, the proportion of studies reporting either an increase or a decrease in anti-inflammatory biomarkers was comparable between the vitreous (increase: 28.5%; decrease: 42.9%) and serum (increase: 20%; decrease: 40%). However, there was a slight difference in the proportion of studies that reported no change in anti-inflammatory biomarkers between vitreous (28.5%) and serum (40%). While all studies carried out on the vitreous found that chemotactic biomarkers increased in PDR, only 71.4% of studies investigating serum levels reported an increase in these markers, relative to control, with the remaining 28.6% reporting no change. No studies measured cell growth markers in the vitreous while 66.7% of studies reported no change in serum epidermal growth factor (EGF) [49, 53] and 33.3% found a decrease in serum insulin-like growth factor-1 (IGF-1) [45] in DR patients relative to controls.

### VEGF

Being a key cytokine that exacerbates aberrant vessel proliferation and permeability in late-stage DR as well as being the target of anti-VEGF agents, studies reporting vitreous and serum VEGF changes with DR severity were also examined (Table 3). Kaviarasan et al*.*, 2015 [40] and Zhou et al*.*, 2012 [58] reported a significant rise in vitreous VEGF levels in PDR patients compared to controls, while no studies reported vitreous VEGF levels in earlier disease stages. Nalini et al*.*, 2017 [51], and Ozturk et al*.*, 2009 [53] reported rising serum VEGF levels as the disease progressed, and found a significant elevation in PDR compared to NPDR patients. Koleva-Georgieva et al*.*, 2011 [48] found no significant change between control, NDR and NPDR groups, but found a significant surge in serum VEGF levels when the disease progressed to PDR. Chorostowska-Wynimko et al*.*, 2005 [45], Kaviarasan et al*.*, 2015 [40] and Lee et al*.*,2008 [49] found no significant difference in VEGF levels as the disease progressed.

**Table 3:** VEGF in vitreous and serum as DR progresses

	**Author, Year**	**Scale**	**Assay**	**Control (n)**	**T2DM**		
					**NDR (n)**	**NPDR (n)**	**PDR (n)**
Vitreous	***Kaviarasan *et al*., 2015 ***[40]	ICDRDSS	ELISA for VEGF, PEDF, BDNF & LXA4; CBA for other cytokines	33.78 ± 29.24 (18)	NA	NA	**971.75 ± 951.03**^**a**^ (27)
	***Zhou *et al*., 2012 ***[58]	NA	ELISA	16.57 ± 15.04 (20)	NA	NA	**1571.58 ± 957.68** (62)
Serum	***Kaviarasan *et al*., 2015 ***[40]	ICDRDSS	ELISA for VEGF, PEDF, BDNF & LXA4; CBA for other cytokines	960.09 ± 876.6 (27)	660.41 ± 446.25 (27)	590.16 ± 422.26 (30)	960.09 ± 876.6 (30)
	***Koleva-Georgieva *et al*., 2011 ***[48]	ETDRS	ELISA	195.21 ± 128.53 (38)	185.89 ± 141.95 (11)	181.07 ± 117.92 (17)	**487.56 ± 225.20** (11)
	***Chorostowska-Wynimko *et al*., 2005 ***[45]	Ophthalmologist	ELISA	375 ± 27.71 (12)	NA	449 ± 176.67 (12)	NA
	***Nalini *et al*., 2017 ***[51]	Ophthalmologist	Immunoturbidimetry for CRP; ELISA for TNF-α and VEGF	77.38 ± 12.23 (50)	84.91 ± 14.78 (50)	**90.27 ± 14.92** (50)	**106.74 ± 8.91** (50)
	***Lee *et al*., 2008 ***[49]	ETDRS	chemiluminescent immunometric assay	NA	86.41 ± 75.77 (28)	59.43 ± 40.72 (39)	102.88 ± 87.3 (7)
	***Ozturk *et al*., 2009 ***[53]	ICDRDSS	Luminex	100.47 ± 49.66 (28)	137.29 ± 84.45 (31)	**177.07 ± 119.51** (49)	**169.88 ± 109.12** (46)

All results displayed as mean±SD (n). **Bold** indicates significant increase relative to non-diabetic controls

*IL* interleukin, *NDR* no diabetic retinopathy, *NPDR* non-proliferative diabetic retinopathy, *PDR* proliferative diabetic retinopathy, *T2DM* Type 2 diabetes mellitus, *NA* not applicable, ^a^extrapolation from graph as no numerical values provided

### Inflammasome biomarkers IL-1β and IL-18

IL-1β and IL-18 are inflammasome biomarkers as their activation and release are directly regulated by activation of the inflammasome. Their vitreous and serum concentrations as DR severity advances are recorded in Table 4.

**Table 4:** Inflammasome biomarkers in vitreous and serum as DR progresses

IL-1β							
	**Author, Year**	**Scale**	**Assay**	**Control (n)**	**T2DM**		
					**NDR (n)**	**NPDR (n)**	**PDR (n)**
Vitreous	***Chen *et al*., 2018 ***[43]^***a***^	ICDRDSS	ELISA	7 (22)	11 (19)	**15 (20)**	**23 (31)**
	***Zhou *et al*., 2012 ***[58]	NA	ELISA	5.10 ± 5.46 (20)	NA	NA	**60.43 ± 27.12 (62)**
Serum	***Cvitkovic *et al*., 2020 ***[41]	NA	CBA	16.4 ± 1.16 (35)	17 ± 3.03 (15)	**18.6** ± **2.97 (14)**	
	***Koleva-Georgieva *et al*., 2011 ***[48]	ETDRS	ELISA	0.58 ± 1.36 (38)	1.15 ± 2.39 (11)	0.55 ± 0.78 (16)	**4.15 ± 4.11 (11)**
	***Lee *et al*., 2008 ***[49]	ETDRS	chemiluminescent immunoassay		0.94 ± 0.81 (28)	2.06 ± 5.94 (39)	0.79 ± 0.67 (7)
	***Quevedo-Martínez *et al*., 2021 ***[54]	ETDRS	CBA	36.23 ± 6.3 (16)	38.1 ± 6.8 (16)	39.4 ± 4.19 (16)	36.4 ± 5.1 (16)
**IL-18**							
	**Author, Year**	**Scale**	**Assay**	**Control (n)**	**T2DM**		
					**NDR (n)**	**NPDR (n)**	**PDR (n)**
Vitreous	***Chen *et al*., 2018 ***[43]^***a***^	ICDRDSS	ELISA	7 (22)	11 (19)	**15 (20)**	**23 (31)**
Serum	***Khalifa *et al*., 2009 ***[47]	Ophthalmologist	ELISA	256.4 ± 13.2 (20)	**361.2 ± 7.4 (20)**	**395.6 ± 12.5 (20)**	**486.6 ± 14.2 (18)**
	***Chorostowska-Wynimko *et al*., 2005 ***[45]	Ophthalmologist	ELISA	335 ± 93.5 (12)	NA	**453 ± 149 (12)**	NA

All results displayed as mean±SD (n). **Bold** indicates significant increase relative to non-diabetic controls

*IL* interleukin, *NDR* no diabetic retinopathy, *NPDR* non-proliferative diabetic retinopathy, *PDR* proliferative diabetic retinopathy, *T2DM* Type 2 diabetes mellitus, *NA* not applicable, ^a^extrapolation from graph as no numerical values provided

#### IL-1β

Vitreous IL-1β levels were measured by Chen et al*.*, 2018 [43] and Zhou et al*.*, 2012 [58]. Chen et al*.*, 2018 [43] found IL-1β levels gradually increased as DR progresses. While no significant difference was found between control and NDR, there was a significant increase between NDR and NPDR, as well as between NPDR and PDR. Zhou et al.*,* 2012 [58] agreed with Chen et al*.*, 2018 [43] and showed significantly higher vitreous IL-1β levels in PDR compared to controls, however, their study did not measure vitreous IL-1β levels in earlier DR stages. For serum IL-1β, Cvitkovick et al*.*, 2020 [41] demonstrated a tendency for increased IL-1β levels from control to NDR and DR. While the study showed a significant increase in IL-1β in DR compared to control, it did not differentiate NPDR from PDR. Koleva-Georgieva et al*.*, 2011 [48] showed no significant difference in serum IL-1β levels in early stages of DR, but a distinct increase leading to PDR. On the other hand*,* Lee et al*.*, 2008 [49] and Quevedo-Martínez et al*.*, 2021 [54] reported no significant difference in serum IL-1β levels between any of the groups.

#### IL-18

Chen et al*.*, 2018 [43] was the only study to measure vitreous IL-18 in DR patients and reported an increase in vitreous IL-18 levels with DR progression. Specifically, while no significant difference was found in vitreous IL-18 levels between control and NDR, there was a significant increase in PDR compared to NDR and NPDR. Khalifa et al*.*, 2009 [47] and Chorostowska-Wynimko et al*.*, 2005 [45] found significantly higher serum IL-18 in NPDR compared to controls with Khalifa et al*.*, 2009 [47] also demonstrating a significant increase in serum IL-18 in PDR relative to NPDR.

## Discussion

This systematic literature review identified several inflammatory biomarkers in both serum and vitreous of T2DM patients with DR and qualitatively examined changes in their expression levels during DR development. Pro-inflammatory biomarkers, such as IL-6, TNF-α, IL-1β, IL-18 and VEGF, as well as chemotactic biomarkers, such as VCAM-1, ICAM-1, MCP-1/CCL2 and IL-8, were not only significantly increased in the vitreous of PDR patients relative to controls, but were also distinctly elevated in the serum in PDR relative to NPDR patients. In contrast, no clear trend was observed for anti-inflammatory biomarkers in either vitreous or serum. Overall, results demonstrated disruption in the balance between pro- and anti-inflammatory signals in both vitreous and serum in DR patients. Moreover, the similarity in the proportion of studies showing an increase, no change, or a decrease in pro-inflammatory, anti-inflammatory and chemotactic biomarkers in vitreous and serum suggests that serum inflammation correlates with vitreous inflammation in DR patients. While the concordance between vitreous and serum levels of inflammasome biomarkers is important for supporting a correlation between serum inflammasome biomarkers and DR progression, it is not possible to routinely screen the vitreous to identify patients at risk of DR progression due to the invasive nature of a vitrectomy. Therefore, monitoring serum inflammasome biomarker levels, easily achievable through blood tests, could provide an accessible method to identify patients at risk of DR progression. Monitoring serum inflammasome biomarkers could add to existing clinical risk predictors for DR progression which could inform the appropriate retinal photo screening interval and further open up opportunities for the development of new drugs which specifically target the NLRP3 inflammasome pathway.

VEGF is highly implicated in DR primarily due to its dual roles in promoting vascular permeability in DME and neovascularization in PDR [5, 8], which is also the target of anti-VEGF agents, one of the treatments for DR [5, 60]. While the release of VEGF into the vitreous is believed to be induced by retinal ischemia [3, 4], the cause and effect of elevated serum VEGF levels in DR development is not clear. Here, studies by Kaviarasan et al*.*, 2015 [40] and Zhou et al*.*, 2012 [58] showed a significant increase in vitreous VEGF levels in PDR compared to controls, suggesting that VEGF acts locally in the posterior eye during PDR. Interestingly, studies also found increased serum VEGF levels with DR progression, with some even showing statistically significant elevation in serum VEGF levels in PDR relative to NPDR [48, 51]. In fact, Guo et al*.*, 2014 [61] found significantly higher VEGF levels in the serum of patients with severe compared to mild-to-moderate DR and the same trend was found in patients with diabetic nephropathy as well as those with diabetic hypertension, suggesting elevated serum VEGF levels in diabetes are associated with the development of systemic vascular diseases. Hamid et al*.*, 2021 [62] also showed that in patients with stage 3 and 4 diabetic nephropathy, serum VEGF levels were significantly higher in those who also had DR compared to those who did not, implying that a threshold serum VEGF level is potentially required for the onset of DR.

Anti-VEGF agents are the only anti-cytokine treatment used clinically to treat DR; however, this systematic literature review shows that besides VEGF, various other pro-inflammatory and chemotactic biomarkers are also upregulated as DR develops. IL-6, which is significantly increased in the vitreous and serum in DR, has been shown to disrupt the barrier integrity of retinal vessels through inducing VEGF, recruiting microglial cells, downregulating tight junction proteins and increasing endothelial cell apoptosis [63, 64]. Through inhibiting the IL-6 trans-signaling pathway in an early DR mouse model, diabetes-induced oxidative damage was significantly reduced at both systemic and retinal levels [65]. Etanercept, a TNF-α blocker, has also been shown to suppress vascular lesions in DR mouse models [66, 67]. This suggests that VEGF is not the only biomarker responsible for vascular lesions and vessel leak in DR. As large numbers of pro-inflammatory and chemotactic biomarkers are involved in DR, targeting a single cytokine is insufficient to completely resolve inflammation in DR. Furthermore, targeting downstream cytokines does not address the underlying DR pathogenesis. In order to halt the development of DR, it may thus be more efficient to target the upstream mechanisms regulating the release of these inflammatory biomarkers.

The NLRP3 inflammasome is a potential upstream target for future DR therapeutics as it plays a key part of the innate immune system that orchestrates inflammatory cascades and is dysregulated in chronic inflammatory diseases such as in T2DM [21, 68]. Activation of the inflammasome complex results in activation of procaspase-1, which subsequently releases pro-inflammatory cytokines IL-1β and IL-18 which mediate the downstream effects of the inflammasome [21, 68]. This systematic review has highlighted a significant increase in biomarkers of the activated NLRP3 inflammasome, IL-1β and IL-18, in the vitreous as DR develops in T2DM patients, suggesting activation of the inflammasome plays a major role in initiating inflammation in DR. Chen et al*.*, 2018 [43], which specifically investigated the role of the NLRP3 inflammasome in T2DM patients with DR, found a gradual increase in vitreous IL-1β and IL-18 as DR developed, with a significant increase in their levels in NPDR relative to control, and an even larger increase in PDR relative to NPDR. This was supported by Zhou et al*.*, 2012 [58], which also found a significant increase in vitreous IL-1β in PDR compared to control using the same detection assay (ELISA); however, this study did not include vitreous data for earlier stages of the disease. Besides Chen et al*.*, 2018 [43], no other studies have measured vitreous IL-18 in T2DM patients with DR. The only other paper that specifically investigated vitreous inflammasome biomarkers in DR was Loukovaara et al*.*, 2017 [19], which showed significantly increased vitreous caspase-1, IL-18 and VEGF but no change in IL-1β between NPDR and PDR groups. However, this study had to be excluded from this systematic literature review due to mixing data of T1DM and T2DM patients, as well as including PDR patients who had received previous anti-VEGF treatments. On the other hand, serum inflammasome biomarkers were also found elevated in DR development. Cvitkovic et al*.*, 2020 [41] and Koleva-Georgieva et al*.*, 2011 [48] found increased serum IL-1β as DR developed; however, the increase was gradual in the former while abrupt in the later study. In contrast, Lee et al*.*, 2008 [49] and Quevedo-Martínez et al*.*, 2021 [54] showed no significant change in serum IL-1β through different stages of DR development. These studies all graded DR using ETDRS except for Cvitkovic et al*.*, 2020 [41] which did not report the grading method. Despite all showing the highest IL-1β levels in PDR patients, the pattern of increase was inconsistent between studies, which may be due to the different detection assays used (CBA for Cvitkovic et al*.*, 2020 [41] and Quevedo-Martínez et al*.*, 2021 [54], ELISA for Koleva-Georgieva et al*.*, 2011[48] and sandwich chemiluminescent immunoassay for Lee et al*.*, 2008 [49]). IL-18 in the serum was also found increased as DR develops. Compared to controls, Khalifa et al*.*, 2009 [47] showed serum IL-18 increased by 1.5 fold in NPDR compared to control and 1.9 fold in PDR compared to control. Similarly, Chorostowka-Wynimko et al*.*, 2005 [45] showed a 1.4 fold increase in NPDR compared to control. Both studies relied on ophthalmologists for DR grading and used ELISA for detection. Furthermore, Chen et al*.*, 2018 [43] also showed increased levels of IL-1β and IL-18 mRNA, as well as protein expression of inflammasome-associated biomarkers NLRP3, ASC and caspase-1 in peripheral blood mononuclear cells, as DR developed from control to NDR, NPDR and PDR. While only a small number of eligible studies were found, these studies coherently supported our hypothesis that inflammasome activation is increased systemically as DR develops, highlighting the potential of a serum-based screening tool for predicting the onset and progression of DR. The most significant increase in IL-1β and IL-18 was found between NPDR and PDR groups in both vitreous and serum, which was coherent with the findings for pro-inflammatory biomarkers including VEGF. This provides insight on the optimal therapeutic window in which preventative anti-inflammasome interventions should be commenced.

There was a surprising lack of literature investigating the role of the inflammasome in the development and progression of DR as there are already a number of NLRP3 inflammasome inhibitors in research which have been proposed as potential DR therapeutics. This includes MCC950 which directly disrupts the oligomerization of the NLRP3 inflammasome complex [37], and Peptide 5 which acts upstream of the inflammasome [33], as well as minocycline, which reduces the production of reactive oxygen species that can trigger inflammasome activation [69]. In particular, anakinra, a human recombinant IL-1 antagonist, has been shown to significantly reduce the progression of choroidal neovascularization and ameliorated endothelial dysfunction in diabetic animal models [35, 70]. Tonabersat, a connexin43 hemichannel blocker, has proven to block the ATP autocrine feedback loop that activates the inflammasome, thus inhibiting the activation of procaspase-1, preventing IL-1β and IL-18 release as well as reducing astrocytosis and Müller cell activation in human retinal explants cultured in DR conditions [34].

Several other significant gaps in literature were revealed in this review. Firstly, most studies only provided vitreous biomarker levels in PDR and control but not in earlier stages of the disease, making it difficult to determine whether the inflammasome is activated in all T2DM patients or only in patients with manifested DR, and whether there is a significant difference between NPDR and PDR. It would be valuable to have the data of vitreous biomarker levels from NDR and NDPR groups; however, this is generally unethical, given the highly invasive nature of vitrectomy. For the control group, studies have retrieved vitreous from patients requiring vitrectomy due to non-diabetic retinal diseases such as macular hole or epiretinal membranes, which may imply the presence of other systemic conditions and may thus not be true controls. However, it is generally very difficult to collect vitreous samples from patients with both early DR and these other retinal diseases. This further emphasizes the need for serum-based DR screening tests that use systemic inflammasome biomarkers to predict DR progression. Secondly, there is a significant lack of longitudinal study that monitors inflammasome biomarker levels over time as DR develops and progresses. Preciado-Puga et al*.*, 2014 [59] was the only longitudinal study included in this review; however, it only had follow-up data for one year, which is insufficient to observe significant progression in a chronic disease such as DR. Furthermore, the study did not measure IL-1β or IL-18. As longitudinal studies track DR progression of individuals over time and account for inter-patient variabilities, such as age, gender, and baseline inflammation levels, more such studies are required in order to determine whether a causal relationship exists between inflammasome biomarker levels and the development and progression of DR.

There are a few limitations in this systematic literature review. Firstly, data were analyzed qualitatively because a meta-analysis could not be performed due to the heterogeneity in DR grading scales, the detection assays used, the small and uneven sample size as well as the small number of studies included. The differences between detection assays are important to take into account as Luminex and CBA, which quantify results based on fluorescence intensity, are more sensitive compared to an ELISA, a colorimetric assay. Secondly, other types of diabetes besides T2DM were not included as the study was designed with minimal differences between study populations in order to make valid comparisons. Lastly, this systematic review did not account for diabetic patients with systemic co-morbidities other than DR as these conditions may potentially cause an exponential increase in pro-inflammatory biomarker levels, masking the difference between different DR severity levels. The reality is that T2DM patients with severe DR are often diagnosed with other microvascular and macrovascular co-morbidities associated with systemic inflammation. As such, it is likely that systemic inflammation drives the development and progression of DR, further supporting the need to measure serum inflammasome biomarkers to predict DR development and progression.

## Materials and methods

A comprehensive search strategy, including pre-specified keywords used to identify articles that have measured biomarker levels in the vitreous and serum of patients with T2DM with and without DR, is provided in Table S1. This systematic review was conducted in accordance with the PRISMA guidelines (see Table S5). The protocol was prospectively registered on PROSPERO (CRD42020181796) and can be accessed from the PROSPERO website.

### Eligibility criteria

Studies that fulfilled the prespecified inclusion criteria (Table 1) were deemed eligible. As the objective of this systematic literature review focused on activation of the inflammasome in chronic metabolic disease, studies containing subjects with DM types other than T2DM, including T1DM, gestational and secondary DM as well as those with cases under 18 years of age were excluded. Studies were eligible for inclusion regardless of whether the study group was or was not receiving medication for T2DM. Studies with the following factors that could potentially affect biomarker measurements were also excluded: 1) DR patients with other ocular diseases or who received intraocular DR treatments, including pan-retinal photocoagulation, anti-VEGF injections or intraocular corticosteroids, within 3 months of the study period, 2) subjects with DME, which can occur at any stage of DR, 3) subjects with systemic inflammatory diseases concordant with DR. Vitrectomy was only indicated in sight-threatening conditions thus control vitreous samples were from non-diabetic subjects requiring vitrectomy due to non-DR conditions, such as macular hole, spontaneous retinal detachment and epiretinal membrane.

### Search methods

A systematic search was conducted on the 29^th^ of September 2021 to identify all pertinent studies using online electronic databases EMBASE, PubMed and Web of Science. The references of the included studies were also searched to identify further relevant studies. EndNote X9 was used to manage the identified studies and record the eligibility status.

### Study selection

Two authors (CYJK and OOM) independently searched the online databases, screened the studies by titles and abstracts and subsequently identified eligible studies as outlined above. Any discrepancies were resolved through discussion and consultation with the third, independent author (IDR).

### Data collection

Two independent authors (CYJK and OOM) extracted the following information from each study: name of author(s), year of publication, study type, biomarker location, DR grading scale and measurement assay (Table S4). CYJK extracted clinical profiles including age, gender, duration of diabetes and HbA1c% levels (Table S3) which was confirmed by OOM. Any discrepancies were resolved through discussion and consultation with the third, independent author (IDR).

### Risk of bias assessment

A modified version of the NOS was used to assess the quality and risk of bias quantitatively and qualitatively. The specific criteria and scoring system were predefined prior to the study (Table S2). The three domains, 1) selection, 2) comparability and 3) exposure/outcome, were assessed using eight questions. A maximum of one star was awarded for each study in the selection and exposure/outcome domain and a maximum of two stars was awarded in the comparability domain. Signaling questions and elaboration were provided to stringently assess the risk of bias involved in each domain. Biomarker measurement techniques were reviewed to ensure sufficient quality assays were used. A “good” quality study required three or four stars in the selection domain, two or three stars in the exposures/outcomes domain, and one or two stars in the comparability domain. A “fair” quality score required two stars in the selection domain, two or three stars in the outcome/exposure domain, and one or two stars in the comparability domain. A “poor” quality score had zero or one star in the selection domain and outcome/exposure domain as well as no stars in comparability domain.

### Data synthesis and analysis

Changes in the levels of vitreous and serum pro-inflammatory, anti-inflammatory, chemotactic and cell growth biomarkers in DR patients compared to controls were summarized (Table 2). Levels of inflammasome biomarkers, IL-1β and IL-18, as well as VEGF, a prominent downstream DR marker, were extracted to analyze their changes through different stages of DR (Tables 3 and 4). Values were converted to mean ± standard deviation using a previously established formula [71–73].

## Supplementary Information

**Additional file 1: Table S1.** Search strategy using Ovid EMBASE. This search strategy was used to identify literature relevant to this systematic literature review.

**Additional file 2: Table S2.** Modified Newcastle Ottawa Scale (NOS) for quality and risk of bias assessment. The quality and risk of bias of each study was assessed using 8 questions in the domains of selection, comparability, and exposure/outcome.

**Additional file 3: Table S3.** Clinical profile of subjects included in the selected studies. Age, gender, duration of diabetes and HbA1c results from each of the included studies were extracted for comparison.

**Additional file 4: Table S4.** Study characteristics. Table summary of the author, year, study type, biomarker location, DR grading scale and measurement in each study.

**Additional file 5: Table S5.** PRISMA Checklist. A 27-item checklist addressing the completeness of the introduction, methods, results, and discussion sections of a systematic literature review.

## Data Availability

The data used are directly extracted from the studies included in this systematic literature review, which are cited in the tables and the in the text.
