# Supplementary material for: Correlation between the progression of diabetic retinopathy and inflammasome biomarkers in vitreous and serum – a systematic review
Source: BMC Ophthalmol. 2022 May 27;22:238. doi: 10.1186/s12886-022-02439-2 (PMC9145105; doi:10.1186/s12886-022-02439-2)
Supplement: Supplementary file 1 — Additional file 1: Table S1. Search strategy using Ovid EMBASE. This search strategy was used to identify literature relevant to this systematic literature review. [file 12886_2022_2439_MOESM1_ESM.docx]

**Table S1:** Search strategy using Embase <1980 to 2021 September 29>

|  | **Advance Search Terms** | **Results** |
| --- | --- | --- |
| 1 | ("diabetic retinopathy" or "proliferative diabetic retinopathy").mp. [mp=title, abstract, heading word, drug trade name, original title, device manufacturer, drug manufacturer, device trade name, keyword heading word, floating subheading word, candidate term word] | 52107 |
| 2 | (inflammasome or NLRP3 or inflammation).mp. [mp=title, abstract, heading word, drug trade name, original title, device manufacturer, drug manufacturer, device trade name, keyword heading word, floating subheading word, candidate term word] | 1005235 |
| 3 | ("biological marker*" or cytokine* or interleukin* or mRNA).mp. [mp=title, abstract, heading word, drug trade name, original title, device manufacturer, drug manufacturer, device trade name, keyword heading word, floating subheading word, candidate term word] | 1956294 |
| 4 | (pathogenesis or "disease course" or "disease exacerbation" or "disease association").mp. [mp=title, abstract, heading word, drug trade name, original title, device manufacturer, drug manufacturer, device trade name, keyword heading word, floating subheading word, candidate term word] | 1813629 |
| 5 | ("vitreous body" or vitreous or vitreal).mp. [mp=title, abstract, heading word, drug trade name, original title, device manufacturer, drug manufacturer, device trade name, keyword heading word, floating subheading word, candidate term word] | 41238 |
| 6 | (serum or sera or blood or "blood test").mp. [mp=title, abstract, heading word, drug trade name, original title, device manufacturer, drug manufacturer, device trade name, keyword heading word, floating subheading word, candidate term word] | 5428574 |
| 7 | 5 or 6 | 5462411 |
| 8 | 1 and 2 and 3 and 4 and 7 | 305 |
