# Supplementary material for: Correlation between the progression of diabetic retinopathy and inflammasome biomarkers in vitreous and serum – a systematic review
Source: BMC Ophthalmol. 2022 May 27;22:238. doi: 10.1186/s12886-022-02439-2 (PMC9145105; doi:10.1186/s12886-022-02439-2)
Supplement: Supplementary file 2 — Additional file 2: Table S2. Modified Newcastle Ottawa Scale (NOS) for quality and risk of bias assessment. The quality and risk of bias of each study was assessed using 8 questions in the domains of selection, comparability, and exposure/outcome. [file 12886_2022_2439_MOESM2_ESM.docx]

Table S2: Modified Newcastle Ottawa Scale (NOS) for quality and risk of bias assessment.

|  |  | |  | | | | | Criteria Scores | | | | |  |
| --- | --- | --- | --- | --- | --- | --- | --- | --- | --- | --- | --- | --- | --- |
|  | **Selection** | | | | |  | **Comparability** | |  | **Exposure/Outcome** | | |  |
| Study | Exclusion of individuals with systemic and ocular history | Case population representative of the general population | | Selection of control group from the general population | Clear definition for the control group |  | Comparable cases and controls: age-matched, go through the same screening procedure and grading for DR | |  | Name of the assay used to measure each biomarker was clearly stated | Same assay was used to measure levels of biomarkers of both cases and controls | Sensitivity of assays and range of measurement stated or quality of assays evaluated | Overall Quality |
| *Adamiec-Mroczek & Oficjalska-Mlyńczak, 2008 [43]* | * |  | |  | * |  | ** | |  | * | * |  | Fair |
| *Blum et al.,2018 [40]* |  |  | | * | * |  | * | |  | * | * | * | Fair |
| *Chen et al.,2018 [44]* | * |  | |  | * |  | ** | |  | * | * | * | Fair |
| *Chen et al.,2016 [45]* | * |  | |  | * |  | ** | |  | * | * | * | Fair |
| *Chorostowska-Wynimko et al.,2005 [46]* |  |  | | * | * |  | ** | |  | * | * |  | Fair |
| *Cvitkovic et a.,2020 [42]* | * |  | |  | * |  | ** | |  | * | * | * | Fair |
| *Doganay et al.,2002 [47]* | * |  | | * | * |  | ** | |  | * | * |  | Good |
| *Kaviarasan et al,2015 [41]* | * |  | |  | * |  | ** | |  | * | * | * | Fair |
| *Khalifa et al.,2009 [48]* | * |  | | * | * |  | ** | |  | * | * | * | Good |
| *Koleva-Georgieva et al.,2011 [49]* | * |  | | * | * |  | ** | |  | * | * |  | Good |
| *Lee et al.,2008 [50]* | * |  | |  | * |  | ** | |  | * | * | * | Fair |
| *Morita et al.,2010 [51]* | * |  | | * | * |  | ** | |  | * | * |  | Good |
| *Nalini et al.,2017 [52]* | * |  | | * | * |  | ** | |  | * | * |  | Good |
| *Ogata et al.,2007 [53]* |  |  | | * | * |  | ** | |  | * | * | * | Fair |
| *Ozturk et al.,2009 [54]* | * |  | |  | * |  | ** | |  | * | * | * | Fair |
| *Preciado-Puga et al.,2014 [60]* | ***** |  | | ***** | ***** |  | ****** | |  | ***** | ***** | ***** | Good |
| *Quevedo-Martínez et al.,2021 [55]* | ***** |  | | ***** | ***** |  | ****** | |  | ***** | ***** |  | Good |
| *Song et al.,2014 [39]* |  |  | |  | * |  | ** | |  | * | * | * | Poor |
| *Wang et al.,2016 [56]* | * |  | | * | * |  | ** | |  | * | * | * | Good |
| *Yan et al.,2018 [58]* | * |  | |  | * |  | ** | |  | * | * | * | Fair |
| *Zhou et al.,2012 [59]* | * |  | |  | * |  | ** | |  | * | * | * | Fair |

A maximum of one star was awarded for each study in the selection domain and exposure/outcome domain and a maximum of two stars was awarded in the comparability domain. Signaling questions were added to specify the risk of bias involved in each domain. “Good” quality: 3-4 stars in the selection domain, 1-2 stars in the comparability domain and 2-3 stars in the exposures/outcomes domain; “Fair” quality: 2 stars in the selection domain, 1-2 stars in the comparability domain and 2-3 stars in the outcome/exposure domain; “Poor” quality”: 0-1 star in the selection domain or 0 star in comparability domain, or 0-1 star in the outcome/exposure domain.
