# Supplementary material for: Correlation between the progression of diabetic retinopathy and inflammasome biomarkers in vitreous and serum – a systematic review
Source: BMC Ophthalmol. 2022 May 27;22:238. doi: 10.1186/s12886-022-02439-2 (PMC9145105; doi:10.1186/s12886-022-02439-2)
Supplement: Supplementary file 3 — Additional file 3: Table S3. Clinical profile of subjects included in the selected studies. Age, gender, duration of diabetes and HbA1c results from each of the included studies were extracted for comparison. [file 12886_2022_2439_MOESM3_ESM.docx]

Table S3: Clinical profile of subjects included in the selected studies

| ***Author, Year*** | **Age (years), mean ± SD (n)** | | | | **Gender (F:M)** | | | | **Duration of diabetes (years)** | | | | **HbA1c (%)** | | | |
| --- | --- | --- | --- | --- | --- | --- | --- | --- | --- | --- | --- | --- | --- | --- | --- | --- |
|  | Control | NDR | NPDR | PDR | Control | NDR | NPDR | PDR | Control | NDR | NPDR | PDR | Control | NDR | NPDR | PDR |
| ***Adamiec-Mroczek & Oficjalska-Mlyńczak, 2008 [43]*** | 63.00 ± 14.58 (15) | NA | NA | 64.63 ± 8.38 (19) | 9:6 | NA | NA | 12:7 | NA | NA | NA | 12.47 ± 6.75 | 5.56 ± 0.49 | NA | NA | 9.21 ± 2.17 |
| ***Blum et al., 2018 [40]*** | 36.6 ± 7.9 (10) | 64.5 ± 10.8 (10) | 71.4 ± 8.9 (10) | 63.3 ± 11.6 (10) | 5:5 | 4:6 | 3:7 | 4:6 | NA | NA | NA | NA | NA | NA | NA | NA |
| ***Chen et al., 2018 [44]*** | 60.7 ± 7.3 (22) | 58.5 ± 8.3 (19) | 62.8 ± 7.5 (20) | 60.6 ± 5.5 (21) | 9:13 | 12:7 | 11:9 | 9:12 | NA | 7.8 ± 3.9 | 13.2 ± 4.7 | 15.2 ± 3.1 | 5.3 ± 0.7 | 7.8 ± 1.4 | 8.0 ± 0.7 | 12.8 ± 3.6 |
| ***Chen et al., 2016 [45]*** | 61.4 ± 8.2 (51) | 60.2 ± 8.7 (47) | 62.3 ± 7.9 (52) | 60.6 ± 5.1 (53) | 20:31 | 22:25 | 32:20 | 23:30 | NA | 8.7 ± 3.3 | 12.8 ± 3.1 | 15.6 ± 3.9 | NA | 8.9 ± 1.9 | 12.7 ± 3.1 | 14.6 ± 2.4 |
| ***Chorostowska-Wynimko et al., 2005 [46]^#^*** | 66.1 ± 3.8 (12) | NA | 65.8 ± 3.9 (12) | NA | 6:6 | NA | 6:6 | NA | NA | 17.2 ± 3.4 | | | NA | 9.60 ± 0.64 | | |
| ***Cvitkovic et al., 2020[42]*** | 69.9 ± 4.7 (35) | 71.9 ± 6.7 (16) | 73.2 ± 5.1 (14) | | 20:15 | 11:5 | 7:7 | | NA | 8.5 (9.0^$^ | 10.0 (9.0)^$^ | | NA | 6.9 (1.5)^$^ | 7.2 (1.3) ^$^ | |
| ***Doganay et al., 2002 [47]^#^*** | 54.1 ± 2.0 (15) | 50.5 ±1.0 (16) | 55.0 ± 1.2 (18) | 66.6 ± 0.7 (19) | 8:7 | 8:8 | 10:8 | 10:9 | NA | 6.0 ± 0.6 | 9.6 ± 1.1 | 23.7 ± 1.3 | NA | NA | NA | NA |
| ***Kaviarasan et al., 2015 [41]*** | Serum: 44 ± 7 (27)  Vitreous: n=18 | Serum: 51 ± 13 (27) | Serum: 58 ± 10 (30) | Serum: 52 ± 7 (30)  Vitreous: n=27 | 11:16 | 10:17 | 10:20 | 26:4 | NA | 5.12 ± 5.20 | 14.90 ± 8.71 | 15.10 ± 6.68 | 5.67 ± 0.44 | 7.24 ± 0.99 | 7.53 ± 1.49 | 7.51 ± 1.78 |
| ***Khalifa et al., 2009 [48]*** | Age matched 60 – 70 (20 each for all groups) | | | | NA | NA | NA | NA | NA | 12.0 ± 1.8 | | | 6.7 ± 0.7 | 8.7 ± 1.2 | 9.2 ± 0.8 | 10.1 ± 0.7 |
| ***Koleva-Georgieva et al., 2011 [49]*** | 57.5 ± 11.8 (38) | average 59.8 ± 9.7 (NDR 11, NPDR 17, PDR 11) | | | 22:16 | 17:11^@^ | | | NA | NA | NA | NA | NA | NA | NA | NA |
| ***Lee et al., 2008 [50]*** | NA | 58.00 ± 8.59 (28) | 59.87 ± 9.00 (46) | | NA | 15:13 | 22:24 | | NA | 8.96 ± 5.36 | 15.17 ± 6.91 | | NA | NA | NA | |
| ***Morita et al., 2010 [51]*** | 59.0 ± 1.3 (91) | 62.8 ± 0.9 (185)  (NDR 58, NPDR 28, PDR 23) | | | 50:41 | NA | NA | 88:97 | NA | 14.3 ± 0.7 | | | NA | 8.1 ± 0.1 | | |
| ***Nalini et al., 2017 [52]^#^*** | Age matched 40-80 (50 each for all groups) | | | | NA | NA | NA | NA | NA | NA | NA | NA | 5.43 ± 2.4 | 8.42 ± 1.6 | 10.6 ± 0.2 | 12.4 ± 0.6 |
| ***Ogata et al., 2007 [53]*** | 55.3 ± 3.7 (33) | 66.0 ± 2.5 (12) | Mild-moderate NPDR 63.3 ± 2.9 (16)  Severe NPDR  62.8 ± 1.2 (39) | 57.1 ± 1.4 (45) | 14:19 | 58:54 | | | NA | NA | NA | NA | NA | 7.5 ± 0.2 | | |
| ***Ozturk et al., 2009 [54]*** | 64.2 ± 8.22 (28) | 63.9 ± 9.50 (31) | 63.2± 8.08 (49) | 60.9 ± 6.14 (46) | 16:12 | 17:14 | 32:17 | 24:22 | 0 | 9.84 ± 7.13 | 12.54 ± 6.16 | 15.89 ± 6.99 | 5.69 ± 0.53 | 7.95 ± 2.06 | 8.10 ± 1.61 | 8.53 ± 2.11 |
| ***Preciado-Puga et al., 2014 [60]^%^*** | NA | 50.9 ± 6.1 (50) | 52.0 ± 6.8 (66) | 54.2 ± 7.1 (41) | NA | NA | NA | NA | NA | 8.7 ± 4.1 | 10.7 ± 3.8 | 14.1 ± 5.8 | NA | 11.0 ± 2.4 | 11.7 ± 3.0 | 11.5 ± 2.8 |
| ***Quevedo-Martínez et al.2021 [55]*** | 58.7±5.8 (16) | 59.7±11.2 (16) | 59±8.5 (16) | 56.9±9.6 (16) | 11:5 | 8:8 | 10:6 | 9:7 | NA | 10.5±7 | 13.4±8 | 13.9±5.4 | NA | 6.5±0.6 | 7.4±1.0 | 7.8±0.8 |
| ***Wang et al., 2016 [56]*** | 58.75 ± 13.38 (20) | 58.51 ± 12.30 (60) | 61.10 ± 11.37 (40) | NA | 11:9 | 32:28 | 22:18 | NA | NA | 8.0 (3.0- 11.0)^$^ | 14.0 (8.0-20.0)^$^ |  | 5.32 ± 0.31 | 7.97 ± 1.82 | 8.79 ± 2.03 | NA |
| ***Yan et al., 2018 [58]*** | 58.17 ± 7.53 (29) | 61.48 ± 4.45 (29) | 58.17 ± 7.53 (32) | 57.90 ± 6.24 (21) | 14:15 | 13:16 | 15:17 | 10:11 | NA | 4.17 ± 1.75 | 7.66 ± 2.12 | 11.14 ± 3.66 | 5.13 ± 0.75 | 7.31 ± 0.56 | 8.18 ± 1.00 | 9.38 ± 1.97 |
| ***Zhou et al., 2012 [59]*** | 60.12 ± 9.58 (20) | NA | NA | 62.79 ± 8.97 (62) | 12:8 | NA | NA | 32:30 | NA | NA | NA | 12.47 ± 6.25 | 4.72 ± 0.82 | NA | NA | 8.21 ± 3.78 |

All values are given as mean ± standard deviation unless indicated otherwise.

*NDR* no diabetic retinopathy, *NPDR* non-proliferative diabetic retinopathy, *PDR* proliferative diabetic retinopathy, *T2DM* Type 2 diabetes mellitus, *NA not available, M-NPDR* mild to moderate NPDR*, S-NPDR* Severe NPDR

^#^ mean (standard error), ^$^ median (interquartile range), *^@^ incomplete data sum not equal to n of total cases, ^%^ NDR, NPDR and PDR groups are replaced by T2DM patients with no complications, slight and moderate complication and severe complications, respectively*
