# Supplementary material for: Correlation between the progression of diabetic retinopathy and inflammasome biomarkers in vitreous and serum – a systematic review
Source: BMC Ophthalmol. 2022 May 27;22:238. doi: 10.1186/s12886-022-02439-2 (PMC9145105; doi:10.1186/s12886-022-02439-2)
Supplement: Supplementary file 4 — Additional file 4: Table S4. Study characteristics. Table summary of the author, year, study type, biomarker location, DR grading scale and measurement in each study. [file 12886_2022_2439_MOESM4_ESM.docx]

Table S4: Study characteristics

| Author, Year | Study type | Biomarker location | DR grading scale | Assay |
| --- | --- | --- | --- | --- |
| *Adamiec-Mroczek and Oficjalska-Mlyńczak,2008 [43]* | Cross-sectional case-control | Vitreous and Serum | NA | ELISA |
| *Blum et al.,2018 [40]* | Cross-sectional case-control | Serum | Ophthalmologist | ELISA |
| *Chen et al.,2018 [44]* | Cross-sectional case-control | Vitreous | ICDRDSS | ELISA |
| *Chen et al.,2016 [45]* | Cross-sectional case-control | Serum | ICDRDSS | Luminex |
| *Chorostowska-Wynimko et al.,2005 [46]* | Cross-sectional case-control | Serum | Ophthalmologist | ELISA |
| *Cvitkovic et al.,2020 [42]* | Cross-sectional case-control | Serum | NA | CBA |
| *Doganay et al.,2002 [47]* | Cross-sectional case-control | Serum | ETDRS | chemiluminescent immunometric assay |
| *Kaviarasan et al.,2015 [41]* | Cross-sectional case-control | Vitreous and Serum | ICDRDSS | ELISA for VEGF, PEDF, BDNF & LXA4; CBA for other cytokines |
| *Khalifa et al.,2009 [48]* | Cross-sectional case-control | Serum | Ophthalmologist | ELISA |
| *Koleva-Georgieva et al.,2011 [49]* | Cross-sectional case-control | Serum | ETDRS | ELISA |
| *Lee et al.,2008 [50]* | Cross-sectional case-control | Serum | ETDRS | chemiluminescent immunometric assay |
| *Morita et al.,2010 [51]* | Cross-sectional case-control | Serum | ICDRDSS | ELISA |
| *Nalini et al.,2017 [52]* | Cross-sectional case-control | Serum | Ophthalmologist | Immunoturbidimetry for CRP; ELISA for TNF-α and VEGF |
| *Ogata et al.,2007 [53]* | Cross-sectional case-control | Serum | ICDRDSS | ELISA |
| *Ozturk et al.,2009 [54]* | Cross-sectional case-control | Serum | ICDRDSS | Luminex |
| *Preciado-Puga et al.,2014 [60]* | Longitudinal | Serum | Ophthalmologist | Immunoturbidimetry high density for CRP; EASI systems with intra-assay for TNF-α and IL-6 |
| *Quevedo-Martínez et al.2021 [55]* | Cross-sectional case-control | Serum | ETDRS | CBA |
| *Wang et al.,2016 [56]* | Cross-sectional case-control | Serum | ICDRDSS | Luminex |
| *Yan et al.,2018 [58]* | Cross-sectional case-control | Vitreous and Serum | ICDRDSS | ELISA |
| *Zhou et al.,2012 [59]* | Cross-sectional case-control | Vitreous | NA | ELISA |

*BDNF* brain derived neurotrophic factors, *CRP* C reactive protein, *IFN-γ* interferon gamma, *IL-6* interleukin 6, *LXA4* lipoxin A4, *PEDF* pigment epithelium-derived factor, *TNF-α* tumor necrosis factor-alpha, *VEGF* vascular endothelium growth factor, *CBA* cytometric bead array, *ELISA* enzyme-linked immunosorbent assay, *ICDRDSS* International Clinical Diabetic Retinopathy Disease Severity Scale, *ETDRSS* Early Treatment Diabetic Retinopathy Study Scale, *NA* not available
